# A Bering Sea marine heatwave triggers unprecedented crab hybridization, challenging fisheries management

**DOI:** 10.1073/pnas.2625670123

**Published:** 2026-10-19

**Authors:** Erin J. Fedewa, Emily R. Ryznar, Connor P. Cleary, Jennifer L. Gardner, Shannon M. Hennessey, W. Christopher Long, Jon I. Richar, Leah S. Zacher, Michael A. Litzow

**Affiliations:** ^a^ Alaska Fisheries Science Center Shellfish Assessment Program, National Marine Fisheries Service, Kodiak, AK 99615

**Keywords:** marine heatwave, hybridization, fisheries management, causal modeling

## Abstract

Marine heatwaves are increasingly recognized for their capacity to generate ecological surprises, yet the pathways that propagate these unexpected responses are often poorly understood. Here, we document an unanticipated ecological response 7 y after a Bering Sea marine heatwave, in which formerly rare snow crab (*Chionoecetes opilio*) × Tanner crab (*C. bairdi*) hybrid abundance increased 13.4-fold, and the proportion of commercial-sized hybrid males increased 13.1-fold, relative to historical averages. Using causal modeling, we show that the hybrid crab surge was mediated by a heatwave-induced collapse of snow crab rather than direct thermal effects on hybrid survival or increased spatial overlap between mature snow and Tanner crab. Our results reveal an indirect cascading pathway through which an extreme climate event triggered unexpected ecological consequences and unforeseen challenges for fisheries management in the Bering Sea. The dramatic increase in abundance of harvestable hybrid crab, coupled with their exclusion from existing management frameworks, highlights the vulnerability of inflexible management systems to unanticipated ecosystem responses.

Marine heatwaves induce pervasive ecosystem perturbations that destabilize coupled social–ecological systems such as fisheries (1). By subjecting ecosystems to physical conditions far exceeding historical baselines, marine heatwaves can trigger unexpected ecological outcomes driven by unrecognized underlying mechanisms (2). These outcomes may expand ecological understanding but often expose vulnerabilities in rigid management frameworks designed for historical conditions (3).

A 2018–2019 Bering Sea marine heatwave triggered abrupt ecosystem disruptions including the population collapse of snow crab (*Chionoecetes opilio*; 4–6). This collapse ranks among the most significant mass mortality events ever observed in a marine ecosystem (4) and led to repeat closures of a fishery worth hundreds of millions of $US annually. The 2018–2019 heatwave event was part of a long-term borealization trend in the Bering Sea (5) that is expected to favor Tanner crab (*Chionoecetes bairdi*), the more boreal congener (7, 8).

Here, we document an unprecedented increase in the abundance of previously rare snow × Tanner crab hybrids as an additional, unanticipated outcome of the 2018–2019 marine heatwave. Using causal models (9), we identify mechanisms driving the surge in hybrid abundance and demonstrate how this ecological surprise has challenged the existing fisheries management system for *Chionoecetes* crabs in the Bering Sea.

## Results

**Fig. 1.**
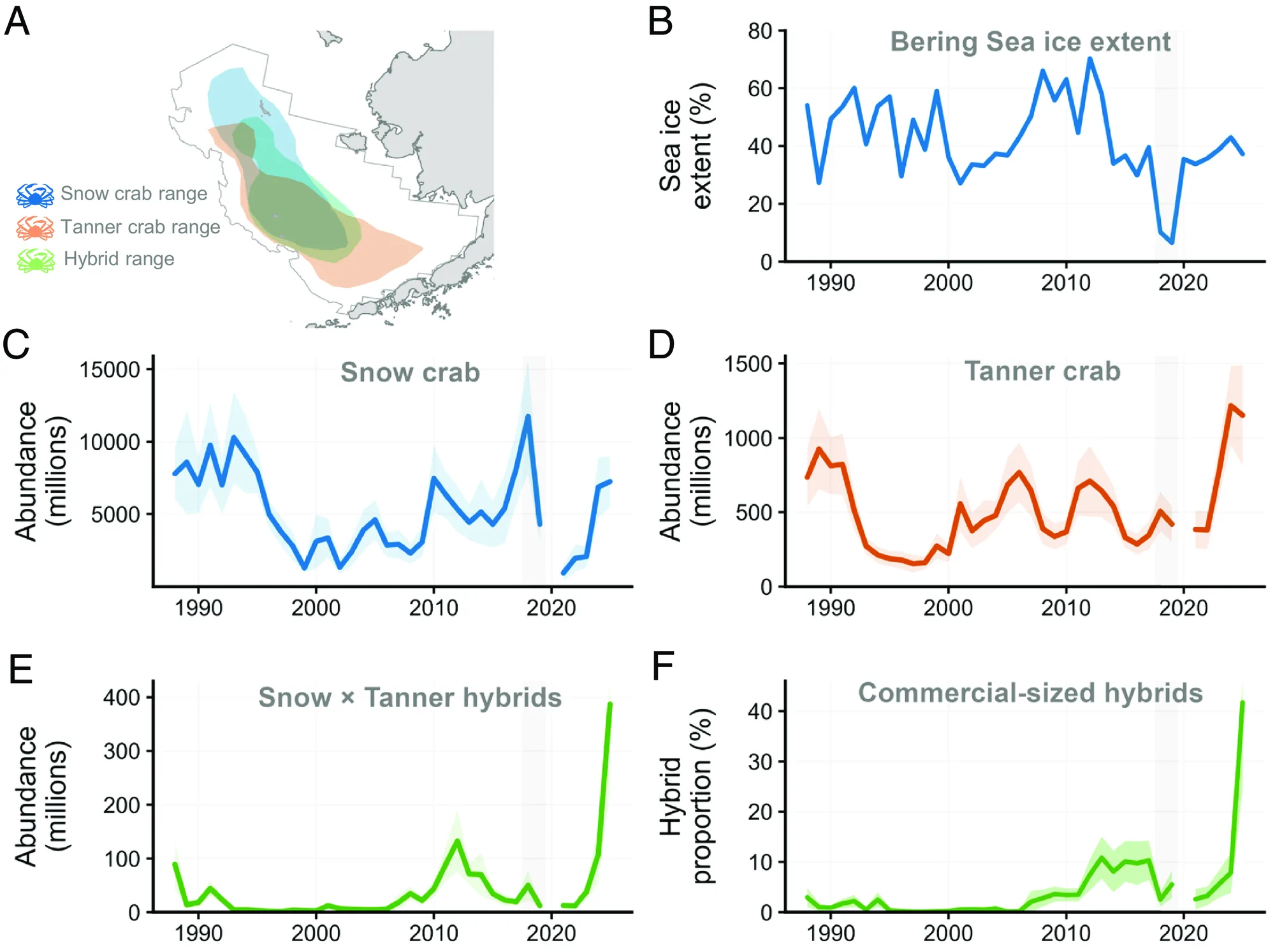
Consequences of the 2018–2019 Bering Sea marine heatwave (vertical gray shading). (*A*) Historical (1988–2025) ranges of snow crab, Tanner crab, and snow × Tanner crab hybrids (50% kernel density estimates) (*B*) Spring sea ice cover. Abundance estimates for (*C*) snow crab, (*D*) Tanner crab, and (*E*) snow x Tanner crab hybrids (±95% CI). (*F*) Proportion of hybrids (±95% CI) within the commercial-sized population of snow and hybrid males.

Snow and Tanner crab ranges in the eastern Bering Sea are shaped by winter sea ice, with a hybrid zone at their boundaries (Fig. 1*A*). The 2018–2019 marine heatwave was characterized by nearly ice-free conditions in March–April (<15% cover; Fig. 1*B*), which triggered the snow crab collapse (4–6). Postheatwave, the snow crab population recovered rapidly (Fig. 1*C*). Tanner crab abundance also increased dramatically to reach a time series high in 2025 (Fig. 1*D*), consistent with the understanding that marine heatwaves may benefit warm-affiliated species (1). Contrary to expectations, the abundance of previously rare snow × Tanner crab hybrids was 13.4-fold greater than the historical mean (1988–2024) in 2025, nearly tripling the prior time-series high (292.6%; Fig. 1*E*).

This unprecedented surge in hybrid crab abundance had immediate consequences for fisheries management. Although legal-sized male hybrids may be harvested as snow crab, their historical rarity has excluded them from the Bering Sea fishery management plan that sets crab harvest quotas. Prior to 2025, hybrids never exceeded 10% of the exploitable male population, but in 2025, nearly half (41.7%) of the commercial-sized males were hybrids (Fig. 1*F*).

**Fig. 2.**
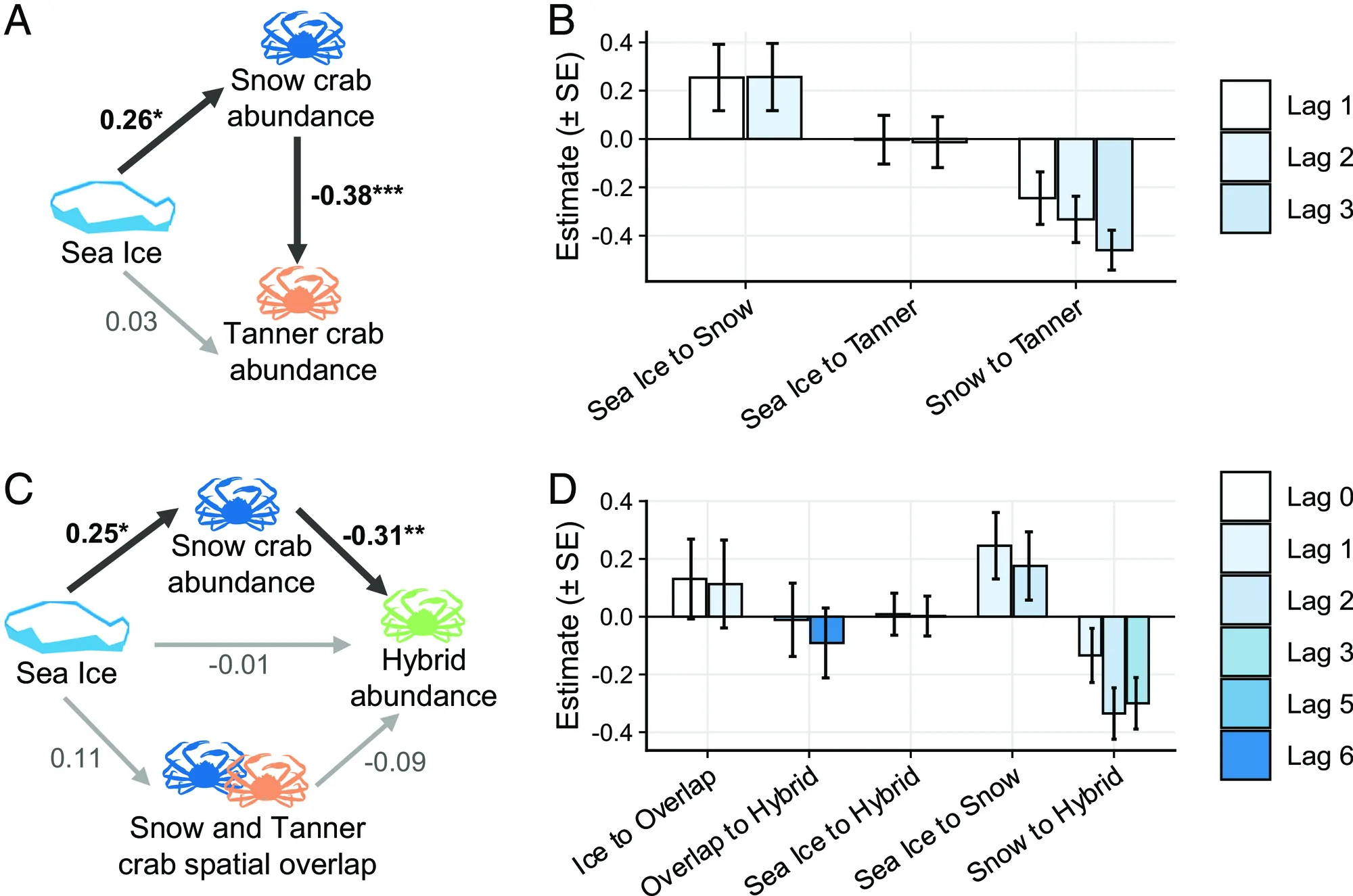
Causal models explaining variable Bering Sea crab abundance. (*A*) Drivers of Tanner crab abundance and (*B*) causal estimates (±SE) across a range of plausible lags. (*C*) Drivers of snow × Tanner crab hybrid abundance, and (*D*) causal estimates across multiple lags. Bold coefficients indicate a *P* value < 0.05 from a two-sided Wald test. *P < 0.05; **P < 0.01; ***P < 0.001.

We used causal inference (Dynamic Structural Equation Models, DSEMs; 9) to evaluate whether increases in Tanner and hybrid crab abundance were linked to the 2018–2019 marine heatwave. Our Tanner crab model showed no support for a direct effect of sea ice extent on Tanner crab abundance (causal estimate = 0.03 ± 0.08 SE, *P* = 0.74, Fig. 2*A*), suggesting that heatwave conditions did not directly promote enhanced survival of the warm-affinity species. However, we did find a significant indirect causal pathway driven by a positive sea ice effect on snow crab abundance (0.26 ± 0.12, *P* = 0.02), which in turn suppressed Tanner crab abundance (-0.38 ± 0.09, *P* < 0.001). These findings are consistent with the expectation that snow crab regulate the northward expansion of Tanner crab through competition or predation (7).

The inability to age *Chionoecetes* crabs, combined with the presence of multiple pseudocohorts in survey abundance estimates, introduces substantial uncertainty into lag specification. We therefore refit the Tanner crab DSEM using a range of biologically plausible lags and found that our results were robust to lag selection (Fig. 2*B*).

Climate warming may accelerate hybridization either by disrupting premating isolation barriers or by altering postmating fitness advantages (10). We used causal inference to differentiate between these two mechanisms to explain the surge in hybrid crab abundance. We did not find support for a premating mechanism (increased spatial overlap between parent species; effect estimate = −0.09 ± 0.12 SE, *P* = 0.45, Fig. 2*C*), nor did we find evidence for a direct sea ice effect as a postmating mechanism (−0.01 ± 0.08 SE, *P* = 0.86). However, we did find strong support for an alternate postmating mechanism, mediated through snow crab abundance (sea ice effect on snow crab abundance: 0.25 ± 0.11, *P* = 0.03; snow crab effect on hybrid crab abundance: −0.31 ± 0.08, *P* < 0.001). The results for our hybrid model were again robust across a range of plausible lags (Fig. 2*D*).

## Discussion

Here, we document a sudden surge in formerly rare snow × Tanner crab hybrids and a dramatic increase in commercial-sized hybrids, highlighting an unanticipated outcome of a marine heatwave. Although ecological surprises often arise from altered species interactions (1), the complex mechanisms underlying these indirect effects are difficult to detect with the correlative approaches commonly used to attribute heatwave impacts (11). Instead, we used a robust causal modeling framework to reveal a previously unrecognized cascading pathway in which a heatwave-induced snow crab collapse drove Tanner and hybrid crab abundance increases through release from competition or predation. This causal pathway accounted for 76% of heatwave effects on hybrid crab abundance, demonstrating the potential for indirect ecological interactions to outweigh direct thermal effects in shaping unexpected heatwave outcomes.

Despite growing recognition that increased hybridization is a potential consequence of climate change (10), this study provides direct evidence linking increased hybridization to a discrete marine heatwave event. Our results suggest that predator or competitive release increased postmating hybrid survival, driving the surge in hybrid crab abundance, rather than a breakdown of premating barriers (10).

Although hybrids can be harvested under snow crab fishery regulations, they are not included as a distinct component of the federal fishery management plan used to establish catch limits. Consequently, there is no regulatory mechanism to account for hybrid abundance when setting harvest quotas, despite hybrids constituting 41% of commercial-sized males targeted by the snow crab fishery. Extensive work on climate change scenarios and adaptation pathways (12) has not included increased hybridization as a plausible outcome of warming. The surprising event we document here does not diminish the value of climate scenario planning but highlights the inevitable limitations of using historical ecological understanding to predict responses to environmental conditions outside the range of historical experience (13). Our results underscore the importance of greater flexibility in management systems (14) to increase resilience amid the unforeseen ecological consequences of marine heatwaves.

## Materials and Methods

Abundance estimates were derived from a fishery-independent trawl survey (1988–2025), where hybrid crab are identified by intermediate morphological traits relative to parent species. Sea ice cover was used as a proxy for marine heatwave conditions because it integrates both direct thermal effects and broader ecosystem changes that influence crab populations (5, 6). To account for cumulative impacts across overlapping pseudocohorts, we fit causal models to Tanner and hybrid crab abundance and evaluated sensitivity to biologically plausible lags. We tested short lags (0 to 2 y) for direct ice effects on survival, intermediate lags (1 to 3 y) for indirect ice effects mediated by snow crab abundance, and longer lags (5 to 6 y) for indirect ice effects mediated by parental species overlap. We refit DSEMs iteratively with each lag to test for consistency in the magnitude and directionality of causal estimates. Causal estimates in Fig. 2 *A* and *C* are for the best model configuration, as determined by marginal AIC.

Detailed methods are provided in the *SI Appendix*, and the data used in these analyses are publicly available through Zenodo (15).

## Supplementary Material

Appendix 01 (PDF)

## Data Availability

Data and code have been deposited in Zenodo (15).

## References

[1] T. Wernberg , Marine heatwaves as hot spots of climate change and impacts on biodiversity and ecosystem services. Nat. Rev. Biodivers. **1**, 461–479 (2025).

[2] D. F. Doak , Understanding and predicting ecological dynamics: Are major surprises inevitable? Ecology **89**, 952–961 (2008). 10.1890/07-0965.1 18481520

[3] C. M. Free , Impact of the 2014–2016 marine heatwave on US and Canada West Coast fisheries: Surprises and lessons from key case studies. Fish Fish. **24**, 652–674 (2023).

[4] C. S. Szuwalski, K. Aydin, E. J. Fedewa, B. Garber-Yonts, M. A. Litzow, The collapse of eastern Bering Sea snow crab. Science **382**, 306–310 (2023). 10.1126/science.adf6035 37856593

[5] M. A. Litzow , Human-induced borealization leads to the collapse of Bering Sea snow crab. Nat. Clim. Change **14**, 932–935 (2024).

[6] E. J. Fedewa, L. A. Copeman, M. A. Litzow, Energetic limitations and mass mortality of Bering Sea snow crab: Interacting effects of warming and density on collapse and recovery. Can. J. Fish. Aquat. Sci. **82**, 1–14 (2025).

[7] J. T. Murphy, Climate change, interspecific competition, and poleward vs. depth distribution shifts: Spatial analyses of the eastern Bering Sea snow and Tanner crab (Chionoecetes opilio and C. bairdi). Fish. Res. **223**, 105417 (2020).

[8] C. Szuwalski , Climate change and the future productivity and distribution of crab in the Bering Sea. ICES J. Mar. Sci. **78**, 502–515 (2021).

[9] J. T. Thorson, A. G. Andrews III, T. E. Essington, S. I. Large, Dynamic structural equation models synthesize ecosystem dynamics constrained by ecological mechanisms. Methods Ecol. Evol. **15**, 744–755 (2024).

[10] A. J. Chunco, Hybridization in a warmer world. Ecol. Evol. **4**, 2019–2031 (2014). 10.1002/ece3.1052 PMC4063493 24963394

[11] H. E. Correia , Best practices for moving from correlation to causation in ecological research. Nat. Commun. **17**, 1981 (2026). 10.1038/s41467-026-69878-z PMC12932701 41730909

[12] A. B. Hollowed , Development of climate informed management scenarios for fisheries in the eastern Bering Sea. ICES J. Mar. Sci. **82**, fsae034 (2025).

[13] J. W. Williams, S. T. Jackson, Novel climates, no-analog communities, and ecological surprises. Front. Ecol. Environ. **5**, 475–482 (2007).

[14] A. S. Golden , Climate adaptation depends on rebalancing flexibility and rigidity in US fisheries management. ICES J. Mar. Sci. **81**, 252–259 (2024).

[15] E. Fedewa , Erin-Fedewa-NOAA/MHW-Emergent-Consequences: Marine heatwave emergent consequences v1.0.0. Zenodo. 10.5281/zenodo.22237476. Deposited 1 September 2026.

