# Supplementary material for: A Bering Sea marine heatwave triggers unprecedented crab hybridization, challenging fisheries management
Source: Proc Natl Acad Sci U S A. 2026 Oct 19;123(43):e2625670123. doi: 10.1073/pnas.2625670123 (PMC13715829; doi:10.1073/pnas.2625670123)
Supplement: Supplementary file 1 [file pnas.2625670123.sapp.pdf]

PNAS

Supporting Information for
A Bering Sea marine heatwave triggers unprecedented crab hybridization, challenging fisheries management

Erin J. Fedewa*, Emily R. Ryznar, Connor P. Cleary, Jennifer L. Gardner, Shannon M. Hennessey, W. Christopher Long, Jon I. Richar, Leah S. Zacher and Michael A. Litzow[1]

Alaska Fisheries Science Center, National Marine Fisheries Service, 301 Research Court, Kodiak, AK 99615, USA

*Corresponding author: Erin Fedewa, 301 Research Court, Kodiak, AK 99615, USA.

[1]Current address: Alaska Coastal Observations and Research, 118 Trident Way, Kodiak, AK 99615, USA

Email: erin.fedewa@noaa.gov

This PDF file includes:

Supporting text
SI References

Extended Materials and Methods

Snow × Tanner Crab Hybrid Biology. Our focal taxon, snow × Tanner crab hybrids, have been documented in the eastern Bering Sea for decades, with individuals exhibiting intermediate morphology and body size relative to the parental species (1). This is the only hybrid crab that is routinely observed on bottom trawl surveys in the Bering Sea. Mature female hybrids show reduced fecundity, as evidenced by relatively small egg clutches and substantial egg mortality (1). Although F2 hybrids and backcrosses have been reported based on genetic markers (2), the genetic evidence for introgression remains inconclusive because alternative explanations, including mutation within parental populations, cannot be excluded (3). Thus, the extent to which hybrids can produce viable offspring and contribute genetically to subsequent generations remains an important uncertainty.

Data Collection. We used annual estimates of snow crab, Tanner crab and snow × Tanner crab hybrid abundance (1988–2025) from the NMFS eastern Bering Sea bottom trawl survey, which occurs during May–July each year. Hybrids were identified using six intermediate morphological traits relative to parent species (4), and expert biologists who staff the surveys cross-train annually to ensure consistency in hybrid identification. However, due to poor detection of small hybrid crab using these morphological traits, we subset abundance datasets to include individuals ≥ 50mm carapace width only.

Bering Sea ice extent was used to characterize marine heatwave conditions in our analyses rather than bottom temperature because sea ice dynamics regulate the formation and persistence of cold bottom waters (5), providing the upstream causal mechanism linking anomalous conditions during the 2018–2019 marine heatwave to thermal effects on crab. We obtained spring sea ice concentration time series (1982–2025) from the ERA5 global reanalysis (6), and spatially subset the dataset to align with the eastern and northern Bering Sea bottom trawl survey grids (approximately 54°N to 66.5°N, 157°W to 178°W). We used March-April sea ice extent because the 2018–2019 Bering Sea marine heatwave was characterized by anomalously low spring sea ice extent, and maximum ice coverage has historically occurred in late March.

Analytical Approach. All analyses and figures were generated using R version 4.5.2. Datasets and script necessary to reproduce our analyses are publicly available and archived on Zenodo (7).

To identify causal drivers of Tanner and hybrid crab abundance, we used dynamic structural equation modeling (DSEM), which estimates simultaneous and lagged effects while handling missing data (8). As a causal inference framework, DSEM offers a robust method for quantifying direct and indirect effects to elucidate underlying mechanisms of species responses to perturbations (9).

First, we developed directed acyclic graphs (DAGs) to identify potential causal pathways linking reduced sea ice extent to shifts in Bering Sea crab populations. We included causal pathways to explicitly test for: 1) direct survival impacts of sea ice on Tanner and hybrid crab abundance, 2) indirect impacts of sea ice on Tanner and hybrid crab abundance mediated by competitive or predatory interactions with snow crab, and 3) indirect impacts of sea ice on hybrid crab abundance mediated by parent species spatial overlap. Spatial overlap between snow and Tanner crab was calculated using Bhattacharyya's coefficient (10), under the assumption that increased overlap of parental species may result in increased interspecies mating and higher hybridization rates (11). Snow × Tanner crab hybrids result from bi-directional parental crosses (3) so we evaluated causal pathways driven by the overlap between functionally mature male snow crab (≥ 101mm carapace width) and mature female Tanner crab, as well as functionally mature male Tanner crab (≥ 103mm carapace width W of 166°, ≥ 113mm carapace width E of 166°) and mature female snow crab. All conditional independence relationships implied by our DAGs were supported ($P = 0.10$), indicating no evidence of model misspecification.

We fit separate DSEM models for Tanner and hybrid crab abundance using the *dsem* R package (8). The general model formulation was:

$$x_t = \Gamma_0 x_t + \Sigma_k \Gamma_0 x_{t-k} + \epsilon_t$$

where x_t is a vector of state variables at time t, Γ_0 is a matrix of simultaneous effects, Σ_k represents lag-k effects among variables, and ϵ_t is a vector of process errors. All state variables were modeled using first-order autoregressive dynamics, and process errors were assumed to follow a multivariate normal distribution. In the Tanner crab DSEM, we included a lag-1 effect of sea ice extent on snow crab abundance, a lag-1 effect of sea ice extent on Tanner crab abundance, and a lag-3 effect of snow crab abundance on Tanner crab abundance. Similarly, in the hybrid crab DSEM we included a lag-1 effect of sea ice extent on snow crab abundance, a lag-1 effect of sea ice extent on hybrid crab abundance, and a lag-2 effect of snow crab abundance on hybrid crab abundance. We also included a lag-0 effect of sea ice extent on parental species spatial overlap, and a lag-6 effect of parental species spatial overlap on hybrid crab abundance. Observation error was fixed (SD = 0.1, corresponding to a 10% coefficient of variation) to improve model convergence and interpretability. To evaluate the robustness of our results to this assumption, we conducted a sensitivity analysis by refitting models across a range of variances (SD = 0.1–0.4). Sensitivity analyses indicated that our causal estimates and corresponding statistical significance remained robust across the range of variance values tested. Tanner and hybrid crab DSEM models used for causal inference showed acceptable diagnostics, with small gradients and invertible Hessian matrices. Estimated latent states closely reproduced observed dynamics for sea ice extent and crab population abundance time series.

Finally, to evaluate parameter identifiability and the ability of the model to recover true path coefficients, we conducted a Monte Carlo simulation-based validation. Data were simulated from fitted DSEM models and then refit to each simulated dataset (n = 1,000 simulations). We compared simulated parameter estimates to their estimated values to assess bias, variance, and overall recovery. Residual diagnostics were also evaluated across simulations to ensure that residuals demonstrated no systematic temporal trends and exhibited minimal autocorrelation across lags. Simulation-based validation of the final Tanner and hybrid crab DSEM models showed strong parameter recovery across all causal pathways and acceptable residual diagnostics, indicating that models adequately capture the temporal dependence structure and the mean dynamics of the system.

SI References

1. A. G. Johnson, Electrophoretic evidence of hybrid snow crab, *Chionoecetes bairdi* × *C. opilio*. *Fish. Bull.* 74, 693–694 (1976).
2. S. E. Merkouris, L. W. Seeb, M. C. Murphy, Low levels of genetic diversity and heterozygosity in Alaskan Tanner crab (*Chionoecetes bairdi*) and snow crab (*C. opilio*) populations. *Fish. Bull.* 96, 69–81 (1998).
3. L. M. Slater, et al., Molecular evaluation of the mating dynamics of snow crab (*Chionoecetes opilio*) in the eastern Bering Sea. Ecol. Evol. 14(10), e11513 (2024).
4. D. Urban, D. Pengilly, L. Jadamec, S. C. Byersdorfer, "Testing carapace morphology characteristics for field identification of *Chionoecetes* hybrids" in Crabs in Cold Water Regions: Biology, Management, and Economics, AK-SG-02-01 (Alaska Sea Grant, University of Alaska Fairbanks, 2002), pp. 117-130.
5. P. J. Stabeno, S. W. Bell, Extreme conditions in the Bering Sea (2017–2018): Record-breaking low sea-ice extent. Geophys. Res. Lett. 46, 8952–8959 (2019).
6. H. Hersbach, et al., The ERA5 global reanalysis. Q. J. R. Meteorol. Soc. 146(730), 1999–2049 (2020).

7. Fedewa EJ, Ryznar ER, Cleary CP, Gardner JL, Hennessey SM, Long WC, Richar JI, Zacher LS, Litzow MA, Marine Heatwave Emergent Consequences v1.0.0. Zenodo. https://doi.org/10.5281/zenodo.22237476 (2026).
8. J. T. Thorson, A. G. Andrews III, T. E. Essington, S. I. Large, Dynamic structural equation models synthesize ecosystem dynamics constrained by ecological mechanisms. Methods Ecol. Evol. 15(4), 744–755 (2024).
9. S. Arif, M. A. MacNeil, Applying the structural causal model framework for observational causal inference in ecology. Ecol. Monogr. 93(1), e1554 (2022).
10. G. Carroll, K. K. Holsman, S. Brodie, et al., A review of methods for quantifying spatial predator–prey overlap. Glob. Ecol. Biogeogr. 28(11), 1561–1577 (2019).
11. A. J. Chunco, Hybridization in a warmer world. Ecol. Evol. 4(10), 2019–2031 (2014).
